# Epidemiology of Anal HPV Infection in High-Risk Men Attending a Sexually Transmitted Infection Clinic in Puerto Rico

**DOI:** 10.1371/journal.pone.0083209

**Published:** 2014-01-06

**Authors:** Vivian Colón-López, Ana Patricia Ortiz, Lizbeth Del Toro-Mejías, Michael Craig Clatts, Joel M. Palefsky

**Affiliations:** 1 Cancer Control and Population Sciences Program, University of Puerto Rico Comprehensive Cancer Center, San Juan, Puerto Rico; 2 Department of Health Services Administration, Graduate School of Public Health, Medical Sciences Campus, University of Puerto Rico, San Juan, Puerto Rico; 3 Department of Biostatistics and Epidemiology, Graduate School of Public Health, Medical Sciences Campus, University of Puerto Rico, San Juan, Puerto Rico; 4 Center for Research on Global Health, Graduate School of Public Health, Medical Sciences Campus, University of Puerto Rico, San Juan, Puerto Rico; 5 University of California San Francisco, San Francisco, California, United States; Georgetown University, United States of America

## Abstract

**Purpose:**

Recent studies in Puerto Rico have reported an increasing incidence of anal cancer in Puerto Rican men. The objective of this study was to determine the prevalence, genotype distribution and risk factors associated with anal HPV infection among men attending an STI clinic in Puerto Rico.

**Methods:**

We conducted a cross-sectional study among 205 men 18 years and older. A comprehensive survey was administered that included a demographic and a behavioral assessment. Separate logistic regression models were performed to determine factors associated with any, high-risk (HR), and multiple anal HPV infection.

**Results:**

The mean age of the study sample was 38.0±13.5 years. The most common HR types were 58, 51 and 31. Overall, HR anal HPV infection was found in 53.5% of the participants. Multiple HPV types in the anal canal were found in 47.6**%** of the sample. A third (29.8%) of participants reported being men who had sex with men (MSM). MSM had a significantly higher prevalence of any, HR and multiple HPV infection (p-value<0.05). Separate multivariate logistic regression analyses showed that being MSM was associated with any (OR = 4.5; [95%CI: 1.9–10.7]), HR (OR = 3.4; [95%CI: 1.1–10.3) and multiple anal HPV infection (OR = 3.6; [95%CI: 1.5–9.1). HIV was marginally associated with multiple anal HPV infection in multivariate analysis (OR = 3.3; 95%CI = 1.0–11.0).

**Conclusions:**

Anal HPV is common among sexually active men attending this STI clinic, with higher likelihood of anal HPV infection among MSM.

## Introduction

Human Papillomavirus (HPV) infection causes approximately 600,000 cases of cancer of the cervix, vulva, vagina, anus and oropharynx annually, as well as benign diseases such as genital warts and recurrent respiratory papillomatosis [1]. HPV is responsible for 100% of the cervical cancer cases and more than 80% of anal cancer cases [2], [3]. Increasing interest in understanding the burden of HPV in men has been documented recently, particularly since the incidence of anal cancer, for which there are no effective screening programs, has been rising over the last couple of decades [4]. In the United States, anal cancer in men has increased from 0.5 per 100,000 in 1974 to 1.3 per 100,000 in 2004 [5]. In Puerto Rico, an increase in percent change of 26.7% among men has been documented, with incidence rates ranging from 1.13 per 100,000 in the period of 1992–1996 to 1.43 per 100,000 in 2009 [6].

High-risk (HR) anal HPV infection, particularly genotypes 16 and 18, is a significant risk factor for the development of anal intraepithelial neoplasia (AIN) and anal cancer [7], [8]. Although a high burden of HPV infection in men attending STI clinics has been documented [9], [10], there are only a small number of studies in these settings that describe the epidemiology of anal HPV, by HPV types. Since a critical first question is to identify which HPV types are prevalent in the anal canal in men attending these clinics, the purpose of this study is to determine the prevalence of anal HPV infection and identify risk factors associated with anal HPV-specific types (any type, HR, and multiple types) in a sample of high-risk men attending an STI clinic in Puerto Rico.

## Methodology

The study was conducted from 2009 to 2011, as part of an ongoing epidemiological study among patients attending a public STI/HIV screening and treatment center in Puerto Rico. This study was previously approved by University of Puerto Rico Medical Sciences Campus (UPR MSC) Institutional Review Board (IRB). The design and methods of the parent epidemiological study have been described elsewhere [11], [12]. Briefly, men and women aged 16 years or older were selected from the clinic waiting room and screened for eligibility (including age and capacity for consent). Participants provided a signed written informed consent for the study procedures and for extraction of selected clinical data from their medical chart. They participated in a face-to-face interview that included demographic, behavioral, history of health services utilization and self-reported STIs.

Following the completion of the behavioral survey, all male participants were invited to participate in the HPV sub-study [13], [14]. In this sub study, men older aged 16 years or older were eligible to participate. Participation involved an additional supplemental survey related to HPV, including assessments of HPV transmission knowledge, perceived susceptibility of HPV-related cancers (penile, anal and oral) and vaccine acceptability. Overall, 206 participants were recruited to participate. Separate informed consents were signed by the study participants after a detailed explanation of the sub-study was provided by research assistants.

### Variables of Interest

A man was considered to have HR HPV infection if he was positive for one or more HR genotypes, whether or not he was also positive for one or more LR HPV genotypes. On the other hand, a man was considered to have LR HPV infection if he was positive only to LR HPV genotypes. Finally, multiple HPV types to HPV were identified if study participant was positive for two or more HR or LR HPV genotypes. Sociodemographic variables included age, education, current employment, annual income and marital status. A man was considered MSM if the participant reported having had sex (anal or oral sex) with another man in his lifetime. Use of cigarettes, alcohol and marihuana during the past 90 days was collected as well. Sexual behaviors included lifetime sexual partners (female and male), lifetime anal sex partners (female and male) and condom use in the last sexual event. Self-reported STIs included HIV and syphilis.

### Genital Specimen Collection

For the anal sample collection, trained clinicians moistened a Dracon swab with tap water and inserted it into the anal canal as far as it would go. Applying gentle pressure to the walls of the anal canal, the physician removed the swab with spiral motion over a 30-second period, and immediately inserted it in a vial containing sample transport medium. After collection, specimens were frozen at the core lab at the Puerto Rico Clinical and Translational Research Consortium (PRCTRC), at the UPRMSC, where they were stored at −70°C; and shipped on dry ice to Dr. Palefsky's laboratory at the University of California at San Francisco (UCSF) for anal HPV typing.

### DNA extraction/HPV detection and typing

DNA was prepared from each STM sample and frozen until the specimens were batched for analysis. To prepare DNA, after the samples were thawed they were heated at 56°C for 1 hour. After cooling, 25 µl of 10 mg/ml proteinase K, (Invitrogen, 250 µg/ml final concentration) were added. The samples were vortexed and digested at 50°C in a waterbath overnight and heated at 95°C for 10 minutes to inactivate PK. After DNA purification, PCR was performed using a modified pool of MY09/MY11 consensus HPV L1 primers as well as primers for amplification of the human beta-globin gene. Five microliters of sample were used for PCR amplification using a 40-cycle protocol. After PCR, 6 µL of amplification mixture were applied to a nylon membrane and probed with a biotin-labeled HPV consensus probe mixture containing HPV 11, 16, 18 and 51 L1 DNA. A separate membrane was probed with a biotin-labeled probe to the human beta-globin gene. Each specimen was also studied for the presence of specific HPV types by preparing membranes as described above with 6 µL of specimen. HPV genotypes sought included HR-HPV types (16, 18, 31, 33, 35, 39, 45, 51, 52, 56, 58, 59, and 66), LR types (6/11, 32/42, 54, 57/2/27, 61, 62, 71, 72, 81, 83, 84, 86/87, 90/106, and 102/89, as well as 2 separate mixtures, mix1 containing 7/13/40/43/44/55/74/91, and mix2 containing 3/10/28/29/77/78/94) and types that are of unknown risk (26/69, 30, 34, 53, 67, 68, 70, 73, 82, 85, 97) [15]. The biological specimen was considered HPV-positive when it was positive with the consensus probes or with one or more probes for specific HPV types. Specimens negative for beta-globin gene amplification were excluded from analysis. Negative controls for each experiment consisted of amplification of solution containing all of the above components except for sample DNA. Positive controls included amplification of cloned HPV DNA and HPV positive cell lines.

### Statistical Analysis

Frequency distributions and descriptive statistics were used to characterize the study sample. Prevalence estimates and 95% confidence intervals (95% CI) for any HPV, HR, LR, and multiple HPV types in the anal canal were calculated. Descriptive statistics were calculated to describe the most prevalent genotypes in this sample. Chi-square analysis or Fisher exact tests were used to evaluate differences in categorical outcome measures. Sociodemographic characteristics were stratified by sexual behavior (MSM vs. Non-MSM). Sexual practices were presented separately by these two groups.

Factors significantly associated with HPV (p<0.05) in the bivariate analysis were included in crude and age-adjusted logistic regression models to identify risk factors associated with anal HPV infection in four separately models: 1) any type, 2) HR, and 3) multiple types as dependent variables). Then, multivariate logistic regression model was performed among all variables that remained significant after adjusting for age. Statistical analyses were performed using SAS (Version 9.1.3, Cory, NC). The 95%CI for prevalence estimations were performed using the Binomial CI calculator from Stata Statistical Software (Release 11, College Station, TX).

## Results

Approximately a third of the study participants visited the clinic for the first time at the time of the interview (28.1%). The principal reasons for which study participants were attending the clinic were evaluation/screening for an STI (not HIV) (61.3%) and HIV screening (32.7%) (*data not shown*).

### Demographic Characteristics

Demographic characteristics of the total sample overall and stratified by sexual behavior MSM vs. Non-MSM are shown in **Table 1**. A third (29.8%) of the study sample reported being MSM. The mean age of the study sample was (38.0±13.5) and (37.7±13.0) years for MSM and Non-MSM, respectively. Bivariate analysis showed significant differences by sexual identity in educational level, employment and annual income (p<0.05).

**Table 1:** Socio-demographic characteristics of a sample of Hispanic men attending an STI clinic in San Juan, Puerto Rico.

Variable	Total Sample (%)	MSM	Non-MSM	p-value
**Age (years)**	38.49±12.73	37.98±13.52	37.65±12.97	0.8755
**Education Level**				
<High School	35 (17.6%)	4 (6.78)	31 (22.30)	<0.0001
High School	72 (36.2%)	14 (23.73)	58 (41.73)	
>High School	92 (46.2%)	41 (69.79)	50 (35.97)	
**Employed**				
No	77 (38.7%)	12 (20.34)	64 (46.04)	0.0007
Yes	122 (61.3%)	47 (79,66)	75 (53.96)	
**Annual Income**				
None	56 (28.1%)	11 (18.64)	45 (32.37)	0.0066
<$15,000	102 (51.3%)	28 (47.46)	73 (52.52)	
≥$15,000	41 (20.6%)	20 (33.90)	21 (15.11)	
**Marital Status**				
Single, never married	116 (58.3%)	13 (25.00)	31 (26.72)	0.4923
Married or cohabitating	52 (26.1%)	29 (55.77)	61 (52.59)	
Separated or divorced	30 (15.1%)	10 (19.73)	24 (20.69)	
**Self-reported STIs**				
HIV	84 (42.21)	40 (67.80)	43 (30.94)	<0.0001
Syphilis	32 (16.16)	22 (37.29)	10 (7.25)	<0.0001

### Sexual behaviors and other practices

Among Non-MSM, the mean age of sexual onset was 15.5±2.6 and the mean number of female sexual partners in their lifetime was 19.3±22.4. More than half (81.0%) reported having had anal sex in their lifetime (receptive or insertive), in which 0.7% reporting having had anal sex in the last 90 days. No condom use in the last sexual event was reported in 43.3% of the non MSM participants (*data not shown*).

Among MSM, 5.9% reported having had sex with women in their lifetime and only 3.4% reporting having vaginal sex with women in the last 90 days. More than half (61.0%) reported having anal sex with a men in their lifetime, with 37.9% reported having had receptive anal sex in the last 90 days. More than half (64.3%) reported having had insertive anal sex with a man during the same period of time. None of the MSM interviewed reported any condom use in the last sexual event (*data not shown*).

### Self-reported STIs

Overall, the prevalence of HIV in this sample was 42.2% [95%CI: 35.3%-49.4%]. Self-reported lifetime prevalence of syphilis was 16.1% [95%CI: 11.3–22.0%]. When stratified by sexual behavior, a higher prevalence of HIV and syphilis was observed among MSM when compared with Non-MSM (p<0.0001) (**Table 1**).

### Anal HPV Prevalence

From the 205 participants from which anal samples were obtained, beta-globin was detected in 192 (93.7%) of the specimens, and thus these are included in this analysis. **Table 2** shows the prevalence of anal HPV infection genotypes. In this sample, 57.8% [95%CI: 50.5%–64.9%] of participants tested positive for any type of HPV. The prevalence of HR anal HPV types was 53.5% [95%CI: 43.0%–65.0%]. The most prevalent HR types were 58, 31 and 51. LR-HPV types were found in 68.6% [95%CI: 57.2%–77.2%] of study participants; the most prevalent LR types were 6/11, 84 and 62. Meanwhile, multiple types were found in 47.6% [95%CI: 36.8–58.7%] of the sample and almost a half (42.7%) of the sample was positive for current HPV vaccine types 6/11, 16 and 18 HPV types.

**Table 2:** Prevalence of Anal Human Papillomavirus (HPV) types (n = 205).

HPV type	N	Percent (%)
**Any HPV type**	**111** *	**57.8 (50.5–64.9)**
*HR HPV*	**46** *	**53.5** **(43.0–65.0)**
16	5	8.1
18	5	8.1
31	7	11.2
33	4	6.5
35	6	9.7
39	3	4.8
45	3	4.8
51	8	12.9
52	1	1.6
56	4	6.5
58	9	14.5
59	1	1.6
66	6	9.7
*LR HPV*	**59** *	**68.6 (57.2–77.2)**
6/11	25	29.7
32/42	3	3.6
54	1	1.2
61	4	4.8
62	7	8.3
71	5	5.9
72	4	4.8
81	4	4.8
83	3	3.6
84	17	20.2
86/87	4	4.8
90/106	6	7.1
Mix 1	1	1.2
*Multiple* **	**41**	**47.6 (36.8–58.7)**
*Undetermined* †	**13**	**15.9 (6.9–22.7)**

Among those sample that could be typified (for any type: n = 192); (for HR and LR types: n = 82).

Multiple HPV types were those who showed positive results in 2 or more HPV types.

Those HPV types that are not classified into high or low risk type.

### Any anal HPV types

**Table 3** shows correlates of any anal HPV infection. In age-adjusted models, men who reported having more than high school education were twice as likely to be positive for any anal HPV type as compared to those men who reported having less than a high school diploma at the time of the interview (age-adjusted OR = 2.1; [95%CI:1.1–3.7]). Those who identified themselves as MSM were more than 6 times more likely to be infected with any anal HPV type (age-adjusted OR = 6.3; 95%CI: 2.9–14.0]) as compared to those men who reported being heterosexual (Table 3). Finally, those participants who reported to be HIV+ were more than 3 times more likely to be infected with any HPV type (age-adjusted OR = 3.4; [95% CI: 1.6–7.4]) compared to HIV- subjects.

**Table 3:** Prevalence and Correlates Associated with Anal HPV Infection (Any Type).

	Any Anal HPV type			
Variable	Prevalence (%)	p-value*	Crude OR (95%CI)	Age-Adjusted OR (95%CI)
*Sociodemographic characteristics*				
**Education Level**				
<High School	11 (36.7%)	0.012	1.00	1.00
High School	36 (55.4%)		2.14 (0.88–5.22)	0.84 (0.45–1.56)
>High School	61 (67.0%)		3.51 (1.48–8.31)	2.05 (1.13–3.73)
**Substance Use (Last 90 days)**				
**Tobacco Use**				
No	23 (51.1%)	0.4505	1.00	
Yes	55 (57.9%)		1.32 (0.65–2.68)	
**Alcohol Use**				
No	39 (52.7%)	0.228	1.00	
Yes	69 (61.6%)		1.44 (0.80–2.61)	
**Drug Use**				
No	65 (54.6%)	0.205	1.00	
Yes	43 (64.2%)		1.49 (0.80–2.76)	
**Marijuana Use**				
No	82 (56.2%)	0.316	1.00	
Yes	26 (65.0%)		1.45 (0.70–3.00)	
**Sexual Identity**				
Non-MSM	58 (45.67%)	<0.001	1.00	1.00
MSM	49 (84.48%)		6.48 (2.93–14.30)	6.34 (2.87–14.01)
*Self-reported STIs*				
**HIV**				
No	53 (50.0%)	0.010	1.00	1.00
Yes	55 (68.75%)		2.20 (1.20–4.04)	3.39 (1.57–7.35)
**Syphilis**				
No	86 (56.21%)	0.191	1.00	
Yes	22 (68.75%)		1.71 (0.76–3.86)	
**Condom Use in Last Sexual Event**				
No	16 (44.44%)	0.7044	1.00	
Yes	21 (40.38%)		0.85 (0.36–2.00)	

Fisher's Exact Test was used.

In multivariate logistic regression models, MSM had 4.49 higher odds of any anal HPV infection after adjusting for level of education and HIV status (OR = 4.5; [95%CI: 1.9–10.7]). No other variables achieved statistical significance in multivariate analysis (*Data not shown*).

### HR HPV types

Participants who reported being MSM were more than 3 times more likely of having HR anal HPV (age-adjusted OR = 3.1; [95%CI: 1.2–8.1]) as compared with those who identified as non-MSM. On the other hand, those subjects who reported having had syphilis in their lifetime were almost 4 times more likely to be infected with HR HPV (age-adjusted OR = 3.8; [95%CI: 1.1–12.9]) as compared to those who have been never diagnosed with syphilis (**Table 4**).

**Table 4:** Prevalence and Correlates Associated with High Risk Anal HPV Infection.

	High Risk Anal HPV type			
Variable	Prevalence (%)	p-value*	Crude OR (95%CI)	Age-Adjusted OR (95%CI)
*Sociodemographic characteristics*				
**Education Level**				
<High School	5 (62.5%)	0.5652*	1.00	
High School	17 (65.4%)		1.13 (0.22–5.86)	
>High School	24 (52.2%)		0.66 (0.14–3.1)	
*Substance Use (Last 90 days)*				
**Tobacco Use**				
No	11 (73.3%)	0.1386	1.00	
Yes	21 (51.2%)		0.38 (0.10–1.40)	
**Alcohol Use**				
No	18 (56.3%)	0.8535	1.00	
Yes	21 (58.3%)		1.44 (0.80–2.61)	
**Drug Use**				
No	30 (58.8%)	0.7508	1.00	
Yes	16 (55.2%)		0.86 (0.34–2.16)	
**Marijuana Use**				
No	39 (60.9%)	0.2135	1.00	
Yes	7 (43.8%)		0.50 (0.17–1.51)	
**Sexual Identity**				
Non-MSM	15 (41.20%)	0.0210	1.00	1.00
MSM	30 (%)		3.23 (1.28–8.18)	3.14 (1.22–8.08)
*Self-reported STIs*				
**HIV**				
No	16 (47.1%)	0.1043	1.00	1.00
Yes	30 (65.2%)		2.11 (0.85–5.22)	2.79 (0.95–8.21)
**Syphilis**				
No	30 (50.0%)	0.0188	1.00	1.00
Yes	16 (80.0%)		4.0 (1.20–13.37)	3.80 (1.13–12.85)
**Condom Use in Last Sexual Event**				
No	5 (50.00)	0.5800	1.00	
Yes	5 (38.46)		0.63 (0.12–3.32)	

Fisher's Exact Test was used.

In multivariate logistic regression models after adjusting for age, level of education and syphilis, MSM had higher odds of HR anal HPV infection (OR = 3.35; 95%CI = 1.1–10.3) (*Data not shown*).

### Multiple HPV Infection

The prevalence of multiple HPV infection was higher among those men who identified as MSM as compared with those who identified as non-MSM (age-adjusted OR = 5.0; 95%CI: 2.1–11.9)]. Finally, those participants who self-reported as HIV+ had more than 2 times increased odds of being infected with multiple HPV types compared to HIV- men (age-adjusted OR = 2.8; [95%CI: 0.95–8.2] (**Table 5**). In multivariate logistic regression models, after adjusting for age and level of education a higher likelihood of multiple HPV infection were observed for MSM (OR = 3.70; 95%CI: 1.36–10.07) and HIV positive men (OR = 3.34; 95%CI = 1.02–10.97) (*Data not shown*).

**Table 5:** Prevalence and Correlates Associated with Multiple Anal HPV Infection.

	Multiple Anal HPV type			
Variable	Prevalence (%)	p-value*	Crude OR (95%CI)	Age-Adjusted OR (95%CI)
*Sociodemographic characteristics*				
**Education Level**				
<High School	6 (54.6%)	0.1100	1.00	
High School	9 (25.0%)		0.28 (0.07–1.13)	
>High School	26 (42.6%)		0.62 (0.17–2.25)	
*Substance Use (Last 90 days)*				
**Tobacco Use**				
No	6 (26.1%)	0.3800	1.00	
Yes	20 (36.4%)		0.38 (0.10–1.40)	
**Alcohol Use**				
No	17 (43.6%)	0.3650	1.00	
Yes	24 (34.8%)		1.09 (0.44–2.69)	
**Drug Use**				
No	25 (38.5%)	0.8956	1.00	
Yes	16 (37.2%)		0.95 (0.43–2.10)	
**Marijuana Use**				
No	33 (40.2%)	0.3857	1.00	
Yes	8 (30.8%)		0.66 (0.26–1.69)	
**Sexual Identity**				
Non-MSM	12 (20.7%)	0.0001	1.00	1.00
MSM	28 (57.1%)		5.11 (2.18–11.97)	5.02 (2.12–11.91)
*Self-reported STIs*				
**HIV**				
No	13 (24.5%)	0.0047	1.00	1.00
Yes	28 (50.91%)		3.19 (1.41–7.24)	2.79 (0.95–8.21)
**Syphilis**				
No	30 (34.9%)	0.1923	1.00	
Yes	11 (50.0%)		1.87 (0.73–4.81)	
**Condom Use in Last Sexual Event**				
No	2 (12.50%)	0.8749	1.00	
Yes	3 (14.29%)		1.17 (0.17, 7.96)	

Fisher's Exact Test was used.

## Discussion

To our knowledge, this is the first epidemiological study that reports the distribution and correlates of anal HPV infection in Puerto Rican men. In this group of high-risk men attending an STI clinic in Puerto Rico, in which the prevalence of HIV infection was high (42.2%), the prevalence of anal HPV infection (any type) was common (57.8%). HR HPV was also common in this sample (54.1%), with similar prevalence for HPV-16 (6.1%) and HPV-18 (6.1%). Similar findings were reported in other studies. For example, a study in Spain, in an STI clinic reported that the prevalence of any HPV type was similar to our study (49.6%) [16]. In an STI unit in Italy, in which 89.4% of the men reported themselves as being MSM [17], the prevalence of any HPV type among men was 92.1% in the anal canal. As expected, a higher overall any HPV type prevalence has been observed among studies performed in STI clinics. However, comparisons with other studies show that findings of anal HPV infection in STI clinics are quite variable, primarily due to different anal sampling collection methods, such as the use of different sampling devices, as well as differences in the characteristics of the populations sampled (e.g. MSM, HIV+) which could account for the varying prevalence of anal HPV in STI settings [18], [19].

After multivariate logistic regression models, higher odds of any, HR and multiple anal HPV infection was reported among MSM. In a population-based study in Puerto Rico, it was documented that MSM have significant higher sexual risk practices as compared to Non-MSM [20]. Since recent reports have highlighted that this group had also higher odds of HIV infection [21], this group might be at increased risk of HPV associated diseases such as anal dysplasia and anal cancer [22]. In our study, multivariate logistic regression analysis showed a marginal association between multiple HPV infection and HIV. However, our study did not found a significant association between HIV and any, or HR anal HPV infection in multivariate analysis. This might be due to the lack of heterogeneity in our sample, in which a high prevalence of HIV was reported.

In Puerto Rico, a 26.9% increase in anal cancer incidence rates has been documented for men [6]. Also, it has been documented that Hispanics with AIDS in Puerto Rico consistently showed a greater risk of AIDS and non-AIDS related cancers compared to the general population of Puerto Rico, and that has not changed over time [23]. Therefore, this information along with the present report merits the development of further investigation in the area of anal HPV infection, anal intraepithelial lesions, and anal cancer, primarily among MSM. Finally, research efforts need to be aligned with capacity building efforts, primarily among health care professionals in STI clinics. Capacity building activities in HPV associated diseases and long-term sequelae needs to be developed among physicians who routinely treat men in STI clinics.

Our findings need to be interpreted with caution. Since this study was done in an STI clinic setting, our findings are not generalizable to the general population of PR. Finally, due to our small sample size and since little variability regarding sexual practices was observed, the lack of identification of well-known predictors of HPV infection (such as HIV infection) were not reported in this sample as an independent predictor of anal HPV infection after multivariate analysis. Also, other clinical factors such as HIV plasma viral load, CD4+ count, were not collected as part of this study. Regarding the HPV laboratory analysis, it is always possible that some infections were missed using one set of primers and probes. This might be possible for all HPV types. Despite these limitations, our study documents for the first time evidence of the high prevalence of anal HPV infection in a clinic-based sample of men in Puerto Rico. Efforts in primary and secondary prevention should target high-risk men in STI clinics, primarily MSM.
